# Multiple Myeloma-Derived Extracellular Vesicles Modulate the Bone Marrow Immune Microenvironment

**DOI:** 10.3389/fimmu.2022.909880

**Published:** 2022-07-07

**Authors:** Raquel Lopes, Joana Caetano, Filipa Barahona, Carolina Pestana, Bruna Velosa Ferreira, Diana Lourenço, Ana C. Queirós, Carlos Bilreiro, Noam Shemesh, Hans Christian Beck, Ana Sofia Carvalho, Rune Matthiesen, Bjarne Bogen, Bruno Costa-Silva, Karine Serre, Emilie Arnault Carneiro, Cristina João

**Affiliations:** ^1^ Myeloma Lymphoma Research Group, Champalimaud Experimental Clinical Research Programme, Champalimaud Foundation, Lisbon, Portugal; ^2^ Faculty of Medicine, University of Lisbon, Lisbon, Portugal; ^3^ Hemato-Oncology Department, Champalimaud Foundation, Lisbon, Portugal; ^4^ Faculty of Medical Sciences, NOVA Medical School (NMS), Lisbon, Portugal; ^5^ Centre of Statistics and Its Applications, Faculty of Sciences, University of Lisbon, Lisbon, Portugal; ^6^ Faculty of Medicine, University of Coimbra, Coimbra, Portugal; ^7^ Neural Plasticity and Neural Activity Laboratory, Champalimaud Experimental Clinical Research Programme, Champalimaud Foundation, Lisbon, Portugal; ^8^ Radiology Department, Champalimaud Foundation, Lisbon, Portugal; ^9^ Centre for Clinical Proteomics, Department of Clinical Biochemistry and Pharmacology, Odense University Hospital, Odense, Denmark; ^10^ Computational and Experimental Biology, Chronic Diseases Research Centre (CEDOC); NOVA Medical School (NMS), Lisbon, Portugal; ^11^ Institute of Immunology, University of Oslo and Oslo University Hospital, Oslo, Norway; ^12^ Systems Oncology, Champalimaud Physiology and Cancer Programme, Champalimaud Foundation, Lisbon, Portugal; ^13^ Molecular Medicine Institute-Laço Hub, Instituto de Medicina Molecular João Lobo Antunes, Lisbon, Portugal

**Keywords:** MOPC315.BM cells, multiple myeloma, mouse model, tumor immune microenvironment, extracellular vesicles

## Abstract

Multiple myeloma (MM), the third most frequent hematological cancer worldwide, is characterized by the proliferation of neoplastic plasma cells in the bone marrow (BM). One of the hallmarks of MM is a permissive BM microenvironment. Increasing evidence suggests that cell-to-cell communication between myeloma and immune cells *via* tumor cell-derived extracellular vesicles (EV) plays a key role in the pathogenesis of MM. Hence, we aimed to explore BM immune alterations induced by MM-derived EV. For this, we inoculated immunocompetent BALB/cByJ mice with a myeloma cell line, MOPC315.BM, inducing a MM phenotype. Upon tumor establishment, characterization of the BM microenvironment revealed the expression of both activation and suppressive markers by lymphocytes, such as granzyme B and PD-1, respectively. In addition, conditioning of the animals with MOPC315.BM-derived EV, before transplantation of the MOPC315.BM tumor cells, did not anticipate the disease phenotype. However, it induced features of suppression in the BM milieu, such as an increase in PD-1 expression by CD4+ T cells. Overall, our findings reveal the involvement of MOPC315.BM-derived EV protein content as promoters of immune niche remodeling, strengthening the importance of assessing the mechanisms by which MM may impact the immune microenvironment.

## Introduction

Multiple myeloma (MM) is a dynamic and highly heterogeneous hematological malignancy, both at the biological level and at the clinical level. This challenging disease is characterized by an uncontrolled proliferation of plasma cells in the bone marrow (BM) (1). The clinical symptoms presented by MM patients include calcium elevation, renal dysfunction, anemia, bone lytic lesions and immune dysfunction (2). Presently, it is well accepted that MM establishment, evolution, and resistance to treatment largely depends on the crosstalk between myeloma and immune cells, namely, macrophages and T cells (3, 4).

The concept of “intercellular communication pathway” was first introduced by Raposo et al. in 1996, in which extracellular vesicles (EV) were identified as key signaling bodies (5). EV are small particles released by all cells and have gained increased importance due to their key role in both physiological and pathological processes, including cancer (6). For instance, EV were shown to be involved in metastatic processes (7) of both solid and hematological malignancies, including MM (8, 9). In fact, efforts are being undertaken to understand the exact role of these vesicles in MM. Until now, it has been shown that myeloma-derived EV are able to induce MM progression by promoting different oncogenic processes, such as (A) angiogenesis and myeloma extravasation, supported by the action of cytokines and miRNA contained in MM-derived vesicles on endothelial cells (10, 11); (B) bone resorption with osteoclast activation and osteoblast inhibition (12); (C) drug resistance, where downregulation of exosomal miR-16-5p, miR15a-5p, miR-20a-5p, and miR-17-5p showed a positive correlation with resistance to bortezomib (inhibitor of the NF-kB pathway and proteasome) (13); and (D) immune suppression via uptake of MM-derived EV by myeloid-derived suppressor cells, resulting in their expansion, and consequent inhibition of T-cell anti-tumor activity (11, 14). Recently, Laurenzana et al. showed for the first time that MM-derived EV can inhibit normal hematopoietic stem and progenitor cells and dysregulate hematopoiesis. These results suggest that MM-derived EV might have an important impact on stem cells (15). Furthermore, we and others have demonstrated that EV content in MM patients is different from healthy subjects, revealing its significance in the MM setting (16–19). These EV-driven interactions through the transfer of functional molecules, such as miRNA or proteins, have been shown to protect neoplastic plasma cells, impacting the BM immunome (6, 20). However, the direct effect of MM-derived EV on other BM-derived cells, such as on NK, yδ, CD4+ or CD8+ T cells is unclear.

In the present study, we hypothesized that MM-derived EV act as tumor promoters by influencing lymphoid populations within the BM microenvironment through cell-to-cell communication, ultimately creating a supportive niche to promote MM cells’ proliferation and dissemination. To test this, we aimed to systematically characterize the immune profile associated with disease establishment and the specific effect of MM-derived EV on lymphocytes. Additionally, the proteomic content of MM-derived EV was compared with control EV. Our findings demonstrate that indeed, MM-derived EV are able to reprogram the phenotype of lymphocytes, supporting the development of therapeutic approaches able to target the EV in the treatment of MM.

## Materials and Methods

### Ethics Statement

All animal experimentation procedures presented in this study were evaluated and approved by the institutional ethical committee and the national competent authority. Euthanasia was performed by CO_2_ inhalation or by intraperitoneal injection of pentobarbital.

### Cell Culture

The murine MOPC315.BM cell line was a kind gift from Professor Bjarne Bogen (Institute of Immunology, University of Oslo and Oslo University Hospital, Norway). MOPC315.BM cells were tested for mycoplasma contamination and cultured in Minimum Essential Medium Eagle (MEME; Sigma) supplemented with 10% fetal bovine serum (Biowest), 1% L-glutamine (Sigma), 1% non-essential amino acid (Sigma), and 1% of penicillin/streptomycin (Gibco). Cells were maintained in an incubator at 37°C with 5% CO_2_. For MOPC315.BM cell line transfection with GFP, 0.5 × 10^6^ cells were plated with polybrene (Sigma) together with GFP-virus (FUGW; plasmid #14883; Addgene) and left overnight. Transfection was further confirmed by flow cytometry. Conditioned medium for EV isolation was obtained after 2 days of MOPC315.BM cells culture in MEME supplemented with EV-free FBS. EV-free FBS was obtained after FBS ultracentrifugation at 100,000 *g* for 2 h and 20 min.

### MM Mouse Model

Six- to 8-week-old BALB/cByJ female mice were used (Charles River Laboratories) and maintained in a specific pathogen- and opportunist-free (SOPF) (21) facility at Champalimaud Foundation, Lisbon, Portugal.

For tumor cell injection, immunocompetent BALB/cByJ mice were injected intravenously in the tail vein with 1 × 10^6^ viable MOPC315.BM GFP+ cells in 100 μl of PBS. Age- and sex-matched control mice were injected with the same volume of PBS. MOPC315.BM-bearing mice were sacrificed upon developing paraplegia of the hind limbs, and together with control mice.

### Cell Sorting and Flow Cytometry Analysis

Femurs from both MOPC315.BM-bearing mice and control mice were collected and flushed for BM characterization. Erythrocytes were osmotically lysed. Cells were incubated with anti-CD16/CD32 (clone 93; Invitrogen) and labeled with a Fixable Viability stain 700 (BD). Lymphoid cells were stained with the following anti-mouse antibodies from BioLegend: anti-CD335 PerCP-Cyanin5.5 (Nkp46; clone 29A1.4), anti-CD279 BV421 (PD-1; clone 29F.1A12), anti-TCR yδ BV510 (clone GL3), anti-CD4 BV605 (clone GK1.5), anti-CD8a BV650 (clone 53-6.7), anti-CD3 BV711 (clone 17A2), anti-CD27 BV785 (clone LG.3A10), anti-CD152 APC (CTLA-4; clone UC10-4B9), anti-CD45.2 APC-Cy7 (clone 104), anti-CD25 PE-Dazzle 594 (clone PC61), and anti-CD44 PE-Cy7 (clone IM7). A Super Bright Complete Staining Buffer (Invitrogen) was added to the mix. Manual compensation was performed in all experiments and unstained cells were used as control. Cells were analyzed on an LSRFortessa™ X-20 and sorted on a FACS Aria™ (BD). Supervised and unsupervised analyses were performed with FlowJo™ v10.7 Software (BD). From the total, 1,000,000 cells on average were acquired, and for the unsupervised analysis, 10,000 live CD45+ CD3+ NKp46+ cells per sample were used. For the unsupervised flow cytometry analysis, a total of 410,000 cells were used. The Uniform Manifold Approximation and Projection (UMAP) was implemented for dimensionality reduction (22) and the plugin FlowSOM was applied for clustering (23). Cluster explorer was used afterwards to visualize and export the data. Cell gating strategy is described in **Supplementary Figure 1**.

### Histopathology

Serial 5-μm sections of paraffin-embedded mouse femurs and vertebrae were stained with hematoxylin and eosin using standard protocols. In parallel, myeloma cells were stained with both anti-CD56 (NCAM; clone E7X9M; Cell Signal) and anti-CD138 (Syndecan-1; clone 281-2; BioLegend) antibodies. Negative controls without the primary antibodies were included. Analysis was made by a pathologist blinded to the experimental groups.

### Magnetic Resonance Imaging

Fixed lumbar spines were immersed in a 10-mm NMR tube filled with Fluorinert^®^ for ex vivo high-resolution magnetic resonance imaging. Specimens were imaged using a 10-mm Micro5™ in a 16.4-T magnetic resonance imaging scanner (Ascend Aeon™, Bruker). Prior to image acquisition, center frequency, RF calibration, acquisition of B0 maps, and automatic shimming were performed. The following pulse sequences were acquired: Axial T2*-weighted 2D FLASH (TE: 2 ms, TR: 250 ms, number of signal averages: 4, slice thickness: 0.5 mm, in-plane resolution: 0.05 × 0.05 mm, flip angle: 30°) and Sagittal T2-weighted 2D RARE with fat suppression (TE: 8.5 ms, TR: 2,250 ms, RARE factor: 8, number of signal averages: 32, slice thickness: 0.25 mm, in-plane resolution: 0.04 × 0.04 mm). The acquired datasets were processed and analyzed in MATLAB^®^ (MathWorks Inc.).

### RNA Extraction, cDNA Production, and Quantitative PCR

Total RNA was extracted from FACS-sorted cells using the RNeasy Micro Kit (Qiagen) according to the manufacturer’s instructions. Reverse transcription was performed using the High-Capacity RNA-to-cDNA Kit (Applied Biosystems), followed by a pre-amplification using the TaqMan PreAmp Master Mix (Applied Biosystems). β*2 microglobulin* or *Gapdh* were used as endogenous reference on a StepOne™ Real-Time PCR system (Applied Biosystems). The C_T_ for the target gene was subtracted from the C_T_ for endogenous references, and the relative amount was calculated as 2^−ΔCT^. TaqMan Gene Expression Assays (Applied Biosystems) were the following: β*2 microglobulin* Mm00437762_m1; *Gapdh* Mm99999915_g1; *Lag3* Mm00493071_m1; *Havcr2* Mm00454540_m1; *Ctla-4* Mm00486849_m1; *Il10* Mm00439616_m1; *Ifng* Mm01168134_m1; *Tnfrsf4* Mm00442039_m1; *Il1b* Mm00434228_m1; *Prf1* Mm00812512_m1; *Gzmb* Mm00442834_m1.

### EV Isolation and Characterization

The supernatant of mice femurs was obtained by centrifugation at 500 g for 10 min and then at 3,000 *g* for 20 min. Afterwards, EV were isolated by sequential ultracentrifugation using a sucrose density gradient, as previously described (24). EV size and particle concentration (10^8^ particles/ml) were analyzed using the NS300 Nanoparticle Tracking Analysis (NTA) system. MOPC315.BM-derived EV were isolated using the same procedure from MEME culture media conditioned with the MOPC315.BM cell line.

### Transmission Electron Microscopy

Freshly isolated EV were absorbed into a 100-mesh formvar/carbon-coated glow-discharged copper electron microscopy grids for 20 min. EV were fixed with 2% formaldehyde in 0.1 M phosphate buffer for 20 min, washed, and stained with 2% uranyl acetate for 5 min. Transmission electron microscopy analysis was performed on a FEI Tecnai G2 Spirit BioTWIN and data were acquired using an Olympus-SIS Veleta CCD Camera. Transmission electron microscopy images were acquired at *Instituto Gulbenkian de Ciência*, Oeiras, Portugal.

### Conditioning With MOPC315.BM-Derived EV

For mouse conditioning, a titration of the amount of MOPC315.BM-derived EV to define the quantity to be injected in the mice was performed. BALB/cByJ mice received 10 μg of proteins in 100 μl of PBS from MOPC315.BM-derived EV, 3 times per week for 3 weeks, for a total of 9 retro-orbital injections. Age- and sex-matched control mice were injected with the same volume of PBS. After conditioning, mice were either sacrificed or intravenously injected with the MOPC315.BM cell line.

### Mass Spectrometry and Protein Analysis

Isolated EV from cultured MOPC315.BM cells and from the BM supernatant of mice (femurs) were processed by the filter-aided sample preparation (FASP) method. Protein solutions containing sodium dodecyl sulfate and dithiothreitol (DTT) were loaded onto filtering columns (Millipore) and washed exhaustively with 8 M of urea in HEPES buffer (25). Proteins were reduced with DTT and alkylated with iodoacetamide. Protein digestion was performed by overnight digestion with sequencing grade trypsin (Promega).

Peptide samples were analyzed by nano-LC-MSMS (Dionex RSLCnano3000) coupled to an Exploris 480 Orbitrap mass spectrometer (Thermo Scientific) virtually as previously described (26). Mass accuracy was set to 5 ppm on the peptide level and to 0.01 Da on the fragment ions. Carbamidomethyl and N-terminal protein acetylation was included as variable modifications. A maximum of four missed cleavages was used. The mass spectrometry data were searched against all proteins in the mouse UniProt protein database (3AUP000000589, downloaded in September 2020) with concatenation of all sequences in reverse maintaining only lysine and arginine in place. The data were searched and quantified with VEMS (27).

Gene set enrichment analysis was performed using the rodent UniProt database (downloaded in December 2021) in FunRich software (version 3.1.3_March 2017). For the quantitative analysis, the data were examined in R statistical programming language (28). The estimated IBAQ values were preprocessed as described by others (29), considering the removal of common MS contaminants followed by log_2_(*x*+1) transformation, quantile normalization, and abundance filtering to optimize overall Gaussian distribution of the quantitative values.

### Statistical Analysis

Statistical analysis was performed using GraphPad Prism (GraphPad Software) and R statistical software. For quantitative data, the non-parametric Mann–Whitney test was performed to determine differences between the two groups. The existence of differences between the percentage of paraplegia of each group was assessed through the log-rank test. Differently expressed EV proteins between groups were identified using the methodology implemented in the “RankProd” package (30) of the R software. This method provides a non-parametric approach for identifying differentially expressed proteins (up- or downregulated) between different groups based on the estimated percentage of false-positive predictions (pfp), which was set at 0.05. Results presented with the the following p-values: *p ≤ 0.05, **p ≤ 0.01, ***p ≤ 0.001, ****p ≤ 0.0001 were considered statistically significant.

## Results

### MOPC315.BM Cells Induce MM Phenotype in Mice

In 2012, Hofgaard and colleagues developed a novel murine MM cell line, MOPC315.BM, with tropism to the BM. As this model faithfully replicates the major characteristics of human MM disease, including osteolytic disease and high serum monoclonal immunoglobulin protein (31, 32) in immunocompetent mice, we first aimed to validate the model and further characterize the immunome of MOPC315.BM model.

**Figure 1:**
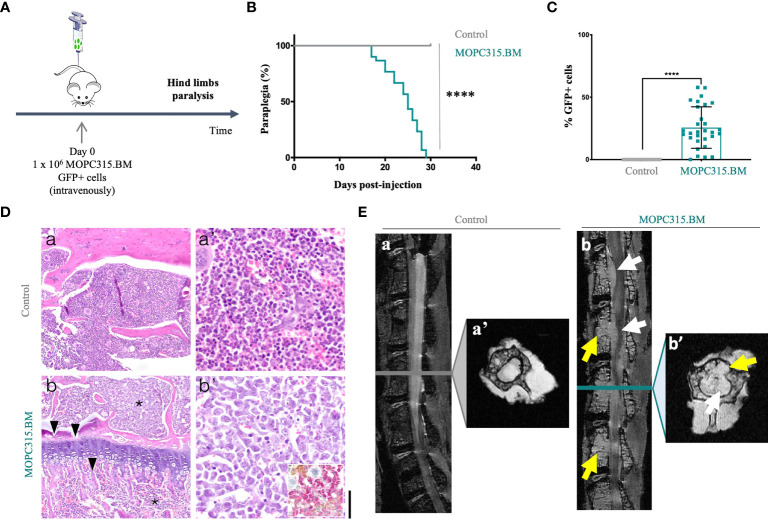
MOPC315.BM cell characterization. **(A)** Schematic representation of the experimental timeline. **(B)** BALB/cByJ mice were injected intravenously with PBS (control; n = 20; gray line) or MOPC315.BM cells (n = 30; blue line). Time to paralysis was calculated through log-rank test evaluation (p < 0.0001). **(C)** Percentage of GFP+ tumor-expressing cells from the BM of mice injected with PBS (control; n = 20; gray squares) and with MOPC315.BM cells (n = 30; blue squares). Statistical analysis was performed using the non-parametric Mann–Whitney test (p < 0.0001). **(D)** Representative histopathological analysis of BM of BALB/cByJ mice; a-a’, control animals show normocellular BM with normal hematopoiesis; b-b’, MOPC315.BM-bearing mice show a hypercellular BM with almost complete replacement of the hematopoietic tissue by a dense monomorphous blast-like round cell population (asterisk), with numerous mitotic figures, large cleaved eccentric nucleus, and with rare giant cells. The myeloma cells are CD138 positive (inset), associated with osteolysis (arrowhead); a, b, original magnification 10× (bar, 150 μm). a’, b’, original magnification 40× (bar, 40 μm). Hematoxylin and eosin; inset, immunohistochemistry for CD56 (DAB; brown) and CD138 (AEC; pink) counterstained with Harris hematoxylin. **(E)** High-resolution magnetic resonance imaging in control (n = 2) versus MOPC315.BM-bearing mice (n = 2). Sagittal fat-suppressed T2-weighted images—a, b; corresponding axial T2*-weighted images through selected vertebral bodies—a’, b’. Representative image reveals extensive BM infiltration in mice with established disease (b and b’, yellow arrows). The tumors disrupt the posterior wall of the vertebral bodies and extend to the spinal canal, causing compression of the spinal cord and involving the nerve roots (b and b’, white arrows). *p*-values are shown as ****p ≤ 0.0001.

In this work, we confirmed that MOPC315.BM cells induced hind limb paraplegia for a median of 25 days post-injection (*p <* 0.0001) (**Figures 1A, B**). Upon phenotype establishment, the percentage of MOPC315.BM GFP+ infiltrating the BM was 25.7% (0.1%–57.8%; *n* = 30) in MOPC315.BM-bearing mice compared to no expression in control mice (*p <* 0.0001) (**Figure 1C**). These percentages underestimate the actual number of MOPC315.BM cells given the initial ratio of 84% (82.7%–88.3%; *n* = 6) of GFP+ cells within the total injected population. We therefore estimate a total infiltration of 30.6% (0.12%–68.8%) of the BM within MOPC315.BM-bearing mice. These results are in accordance with the disease in humans, where a plasma cell infiltration in the BM greater than 10% is used to diagnose MM (33). BM infiltration by malignant plasma cells was further confirmed by histopathological and imaging analysis in MOPC315.BM-bearing mice. Specifically, there was immunoreactivity for CD138 (Syndecan-1) in MM cells infiltrating the long bones and lumbar vertebrae BM. Immunostaining for CD56 (NCAM) was mainly present in endosteal and perivascular niches (**Figure 1D**). Magnetic resonance imaging of lumbar spines of MOPC315.BM-bearing mice showed a clear medullar infiltration with emerging tumors 27 days post-injection (**Figure 1E**). Overall, our results confirmed that hind limb paralysis is associated with MOPC315.BM cell infiltration in the BM, causing tumors leading to spinal cord compression.

### Myeloma Cells Induce a Dual Effect on the BM Lymphocytes

**Figure 2:**
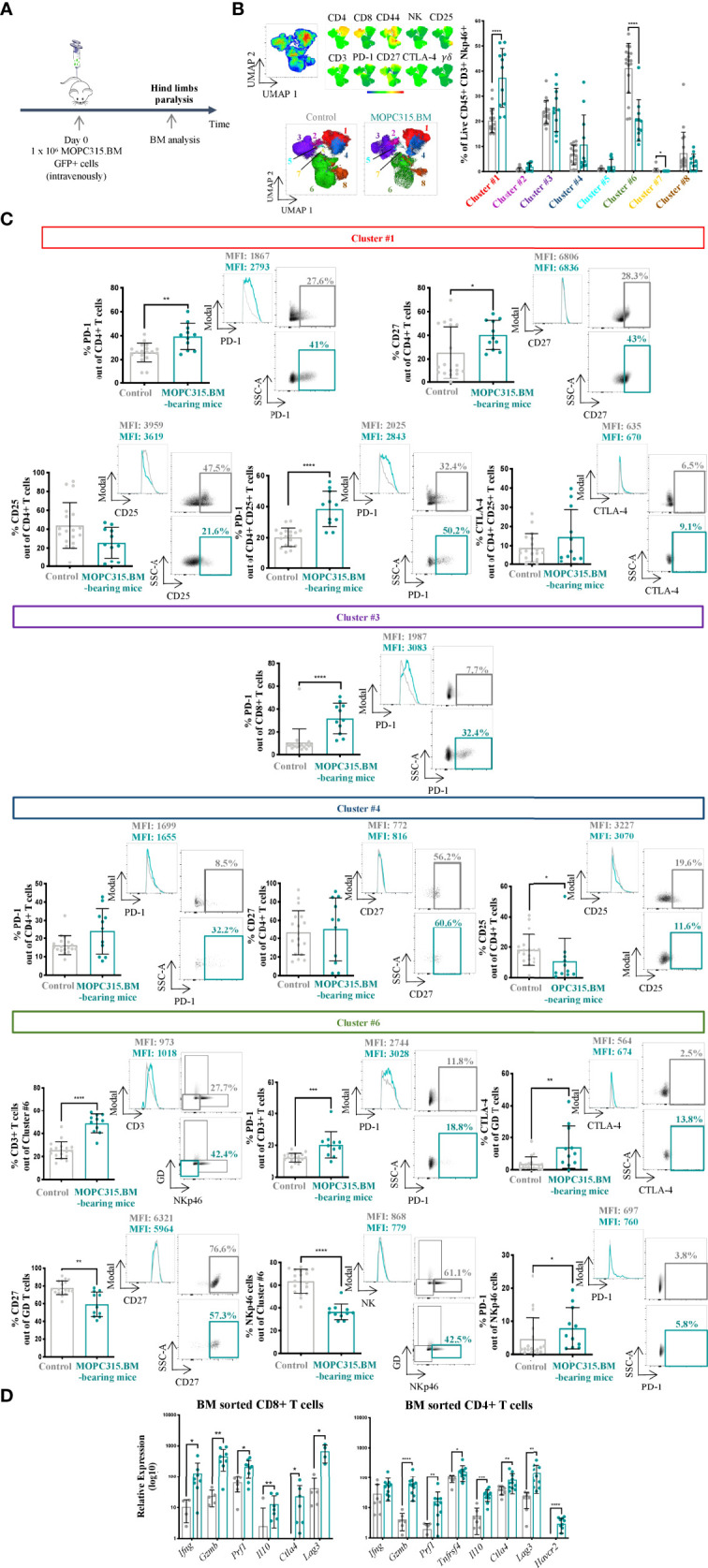
Characterization of the lymphoid compartment upon myeloma’s establishment in the BM. **(A)** Schematic representation of the experimental timeline. **(B)** Unsupervised analysis of lymphoid populations using dimensionality reduction (UMAP). The different color codes represent the intensity of each marker among each cluster on the concatenated live CD45+ CD3+ NKp46+ cells (top left). Representative UMAP of live CD45+ CD3+ NKp46+ cells from control and MOPC315.BM-bearing mice (bottom left). Clustering of 8 cell populations using FlowSOM in control (n = 18; gray) and MOPC315.BM-bearing mice (n = 11; blue) (right). **(C)** Percentage of expression of specific immune markers (left), respective histograms (middle) and dot plots (right) from MOPC315.BM-bearing mice (n = 11; blue) compared to controls (n = 18; gray) from cluster #1 (red), cluster #3 (dark purple), cluster #4 (dark blue), and cluster #6 (green). Controls comprise a pool of three groups: (a) mice injected intravenously in the tail with 100 μl of PBS; (b) mice conditioned 9 times with 100 μl of PBS retro-orbitally injected; and (c) mice conditioned 9 times with 100 μl of PBS retro-orbitally injected and intravenously injected in the tail with 100 μl of PBS. **(D)** Sorted CD8+ and CD4+ T-cell populations for RT-qPCR analysis. MOPC315.BM-bearing mice (n = 4 to 11; blue dots) versus control mice (n = 4 to 8; gray dots). A logarithmization base 10 transformation was applied to better visualize the data. Proven outliers were removed after performing the ROUT method. Statistical analysis was performed using the non-parametric Mann–Whitney test. p-values are shown as *p ≤ 0.05, **p ≤ 0.01, ***p ≤ 0.001, ****p ≤ 0.0001.

To better understand the dynamics of the BM immune niche in this MM mouse model, we performed an immunophenotypic characterization of the lymphoid compartment upon myeloma establishment (**Figure 2A**). For that, NK, yδ, CD4+, and CD8+ T cells were analyzed within the concatenated live CD45+ CD3+ NKp46+ dataset using an unbiased approach. We applied dimensionality reduction using UMAP and ran FlowSOM to obtain 8 different clusters (**Figure 2B**). We found significant differences between MOPC315.BM-bearing mice and control mice in the frequency of cell clusters #1 (*p <* 0.0001), #6 (*p <* 0.0001), and #7 (*p* = 0.0124) (**Figure 2B**). Cluster #1, corresponding to CD4+ T cells, was more represented in MOPC315.BM-bearing mice than in controls (*p <* 0.0001). CD4+ T cells showed an increased expression of the immune checkpoint (IC) PD-1 (*p* = 0.0018) and of CD27 (*p* = 0.0311). We also found a difference in the expression of PD-1 within CD4+ CD25+ cells in MOPC315.BM-bearing mice compared to controls (*p <* 0.0001). A tendency to an increase in the expression of CTLA-4 within CD4+ CD25+ T cells was also identified (**Figure 2C**). Remarkably, although there was no statistical difference between MOPC315.BM-bearing mice and controls within cluster #3 (*p* = 0.7320), we found an increase in CD8+ T cells expressing PD-1 in MOPC315.BM-bearing mice compared to controls (*p <* 0.0001) (**Figure 2C**). A significant decrease in the expression of CD25 by CD4+ T cells in MOPC315.BM-bearing mice compared to controls was detected in cluster #4 (*p* = 0.0108). Despite being not significant, we observed an increase in the expression of PD-1 by CD4+ T cells in MOPC315.BM-bearing mice compared to controls (**Figure 2C**). Regarding cluster #6, it was less represented in MOPC315.BM-bearing mice compared to controls and did not allow the distinction of specific cell subsets, comprising CD3+ (38.7%), yδ + T (12.1%), and NKp46+ cells (49.2%). In this cluster, an increase in PD-1 expression by both CD3+ T cells (*p* = 0.0008) and NKp46+ cells (*p* = 0.0211) and an increase of CTLA-4 in yδ + T cells in MOPC315.BM-bearing mice compared to controls were evident (*p* = 0.0070). This was accompanied by a decrease in γδ + T cells expressing CD27 in MOPC315.BM-bearing mice (*p* = 0.0010) (**Figure 2C**). The phenotype of clusters represented by less than 5% of cells was not analyzed (clusters #2, #5, #7, and #8). Among all the clusters, there were no statistical differences in the expression of CD44 between control and MOPC315.BM-bearing mice (cluster #1, *p* = 0.49; cluster #3, *p* = 0.35; cluster #4, *p* = 0.95; cluster #6 for CD3+ cells, *p* = 0.41; cluster #6 for γδ + T cells, *p* = 0.53; cluster #6 for Nkp46+ cells, *p* = 0.32). For all these comparisons, biologically identical control mice were pooled together using mice (a) injected intravenously in the tail with 100 μl of PBS; (b) conditioned 9 times (3 times a week for 3 weeks) with 100 μl of PBS retro-orbitally injected; and (c) conditioned 9 times (3 times a week for 3 weeks) with 100 μl of PBS retro-orbitally injected and intravenously injected in the tail with 100 μl of PBS. Overall, this immunophenotypic analysis showed an alteration of the immune population representativity, revealing a clear shift on the BM immune microenvironment upon MM establishment towards a more immunosuppressed T lymphocyte microenvironment.

The CD8+ and CD4+ T-cell populations were further analyzed through gene expression quantification using RT-qPCR. CD8+ T cells in MOPC315.BM-bearing mice had higher expression levels of both activation and suppressive markers than in mice previously injected with 100 μl of PBS (controls), including *Gzmb* (*p* = 0.0031) and *Il10* (*p* = 0.0044), respectively (**Figure 2D**, left). A similar observation was made for CD4+ T cells in MOPC315.BM-bearing mice compared to controls, with an increase in the expression of genes involved in both anti- and pro-tumoral processes, namely, *Prf1* (*p* = 0.0046) and *Havcr2* (*p <* 0.0001), respectively (**Figure 2D**, right). Regarding *Ifng*-expressing CD4+ T cells, we detected a trend towards an increased expression in MOPC315.BM-bearing mice compared to controls (*p* = 0.0853).

Altogether, these results suggest that, upon MM establishment, lymphocytes display a dual phenotype in which pro-tumor features are stronger than the anti-tumoral, allowing for myeloma cells to escape BM immune surveillance and successfully expand.

### MOPC315.BM-Derived EV Modulate the BM Immunome

**Figure 3:**
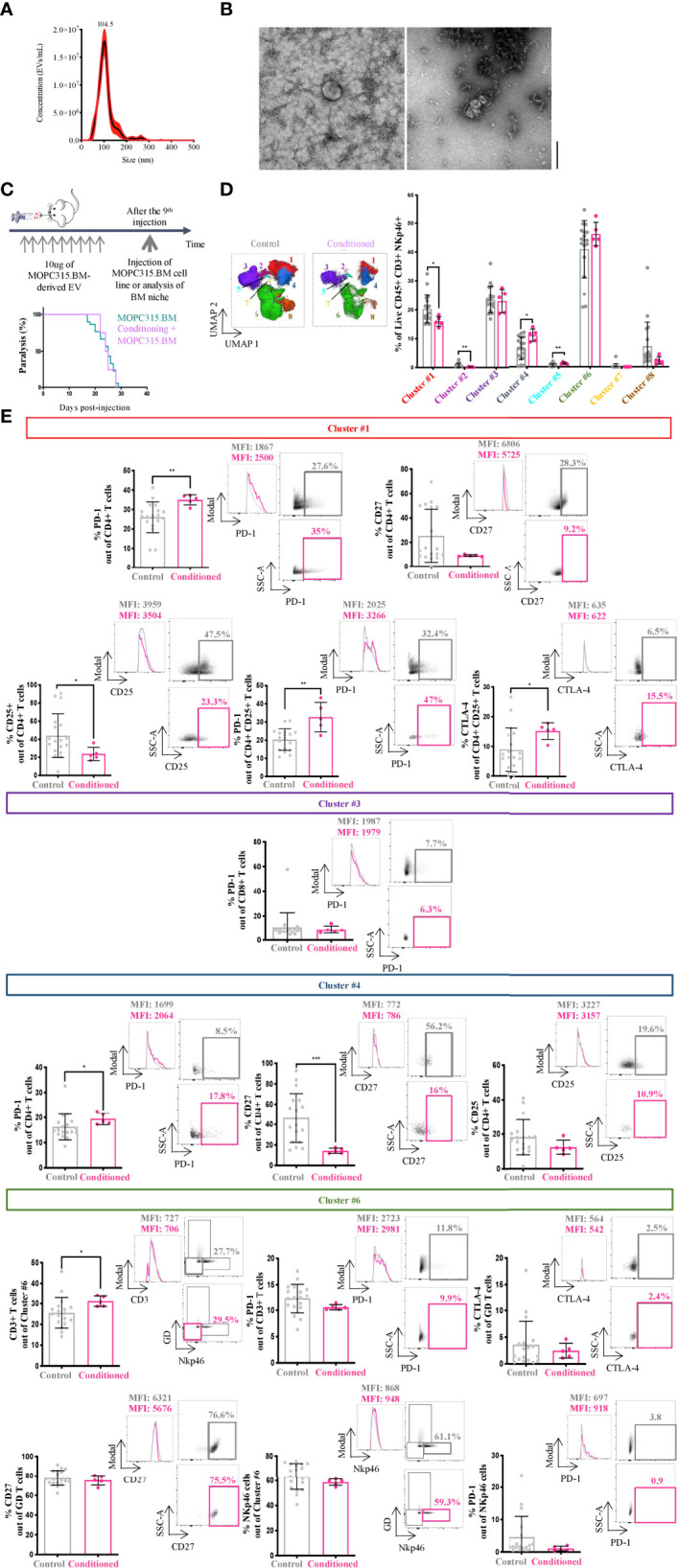
MOPC315.BM-derived EV characterization and BM immune niche characterization. **(A)** Representative NTA curve of MOPC315.BM-derived EV. **(B)** Representative TEM image of MOPC315.BM-derived EV (performed at Instituto Gulbenkian de Ciência, Oeiras, Portugal). Scale bar = 200 nm. **(C)** Schematic representation of the experiment’s timeline (top). Time to paralysis of BALB/cByJ mice conditioned with MOPC315.BM-derived EV followed by MOPC315.BM cells’ injection (n = 8; purple line) compared to mice without conditioning (MOPC315.BM-bearing mice; n = 30; blue line) (bottom). Analysis was performed using the log-rank test (p = 0.85). **(D)** Unsupervised analysis of lymphoid populations using dimensionality reduction (UMAP) on concatenated live CD45+ CD3+ NKp46+ cells in control and conditioned mice before MOPC315.BM cells’ injection (left). Clustering of 8 populations using FlowSOM in control (n = 18; gray) and educated mice (n = 5; pink) (right). **(E)** Percentage of expression of specific immune markers (left), respective histograms (middle) and dot plots (right) from conditioned mice (n = 11; pink) versus controls (n = 18; gray) from cluster #1 (red), cluster #3 (dark purple), cluster #4 (blue) and cluster #6 (green). Controls include a pool of three groups: (a) mice injected intravenously in the tail with 100 μl of PBS; (b) mice conditioned 9 times with 100 μl of PBS retro-orbitally injected; and (c) mice conditioned 9 times with 100 μl of PBS retro-orbitally injected and intravenously injected in the tail with 100 μl of PBS. Statistical analysis was performed using the non-parametric Mann–Whitney test. p-values are shown as *p ≤ 0.05, **p ≤ 0.01, ***p ≤ 0.001, ****p ≤ 0.0001.

We aimed to understand whether MOPC315.BM-derived EV drive disease progression by changing the BM immune microenvironment. For this, isolated MOPC315.BM-derived EV were characterized in terms of both size and concentration by NTA and Transmission electron microscopy, respectively (**Figures 3A, B**). Mice were conditioned with injections of MOPC315.BM-derived EV during 3 weeks prior to intravenous injection of MOPC315.BM cells (**Figure 3C**, top). This time frame corresponds to the median of 25 days until mice develop the expected phenotype.

We observed no alteration on the time to MM phenotype establishment in conditioned mice compared to controls (24.5 days versus 25 days; *p* = 0.85) (**Figure 3C**, bottom). Nonetheless, the characterization of the BM niche by multiparametric flow cytometry before tumor implantation demonstrated a statistically significant shift in the phenotype of lymphocytes between conditioned and control. The most striking differences found in the BM microenvironment were on cell clusters #1 (*p* = 0.0471), #2 (*p* = 0.0037), #4 (*p* = 0.0193), and #5 (*p* = 0.0053) (**Figure 3D**). Cluster #1 presented an increase in PD-1-expressing CD4+ T cells in conditioned versus control mice (*p* = 0.0067) (**Figure 3E**). Although not significant, we also observed a decrease in the expression of CD27 by CD4+ T cells (*p* = 0.1296) (**Figure 3E**). Within cluster #1, 30% of the cells were CD4+ CD25+ (including Tregs). A decrease in their frequency (*p* = 0.0303) and an increase in the IC PD-1 (*p* = 0.0067) and CTLA-4 (*p* = 0.0303) were found (**Figure 3E**). Cluster #4, also corresponding to CD4+ T cells, showed an increase in PD-1 (*p* = 0.0362) and a decrease in CD27 (*p* = 0.0007)-expressing cells in conditioned mice compared to controls (**Figure 3E**). Within cluster #6, we observed an increase in the frequency of CD3+ cells in MOPC315.BM-EV conditioned mice compared to controls (*p* = 0.0158). No statistical differences were found in other analyzed markers, including PD-1-expressing CD3+ T cells (*p* = 0.07; **Figure 3E**). Also, in cluster #3, no statistically significant differences were found between the two groups (**Figure 3E**).

The phenotype of clusters representing less than 5% of cells was not analyzed (clusters #2, #5, #7, and #8). Accounting for all the clusters, there were no statistically significant differences in the expression of CD44 between control and conditioned mice (data not shown; cluster #1, *p* = 0.86; cluster #3, *p* = 0.59; cluster #4, *p* = 0.91; cluster #6 for CD3+ cells, *p* = 0.29; cluster #6 for γδ+ T cells, *p* = 0.28; cluster #6 for Nkp46+ cells, *p* = 0.23). Interestingly, when we compared MOPC315.BM-bearing mice with conditioned MOPC315.BM-bearing mice, an increase in the expression of (1) CD27+ by CD25+ CD4+ T cells in cluster #1 (*p* = 0.0003) (2), CTLA-4-expressing CD4+ T cells in cluster #4 (*p* = 0.0109), and (3) CTLA-4 and CD27 by CD3+ T cells in cluster #6 (*p* = 0.0154; *p* = 0.0003, respectively) was seen. Furthermore, we saw a clear increased expression of the IC PD-1 by NKp46+ cells within cluster #6 in MOPC315.BM-bearing mice compared to conditioned MOPC315.BM-bearing mice (*p* = 0.0204) (data not shown). For all these comparisons, biologically identical controls were used comprising a pool of mice (a) injected intravenously in the tail with 100 μl of PBS; (b) conditioned 9 times (3 times a week for 3 weeks) with 100 μl of PBS retro-orbitally injected; and (c) conditioned 9 times (3 times a week for 3 weeks) with 100 μl of PBS retro-orbitally injected and intravenously injected in the tail with 100 μl of PBS. Altogether, these results strongly suggest the involvement of MOPC315.BM-derived EV in immune niche modifications, promoting a suppressive milieu.

### Proteomic Analysis of MOPC315.BM-Derived EV

**Figure 4:**
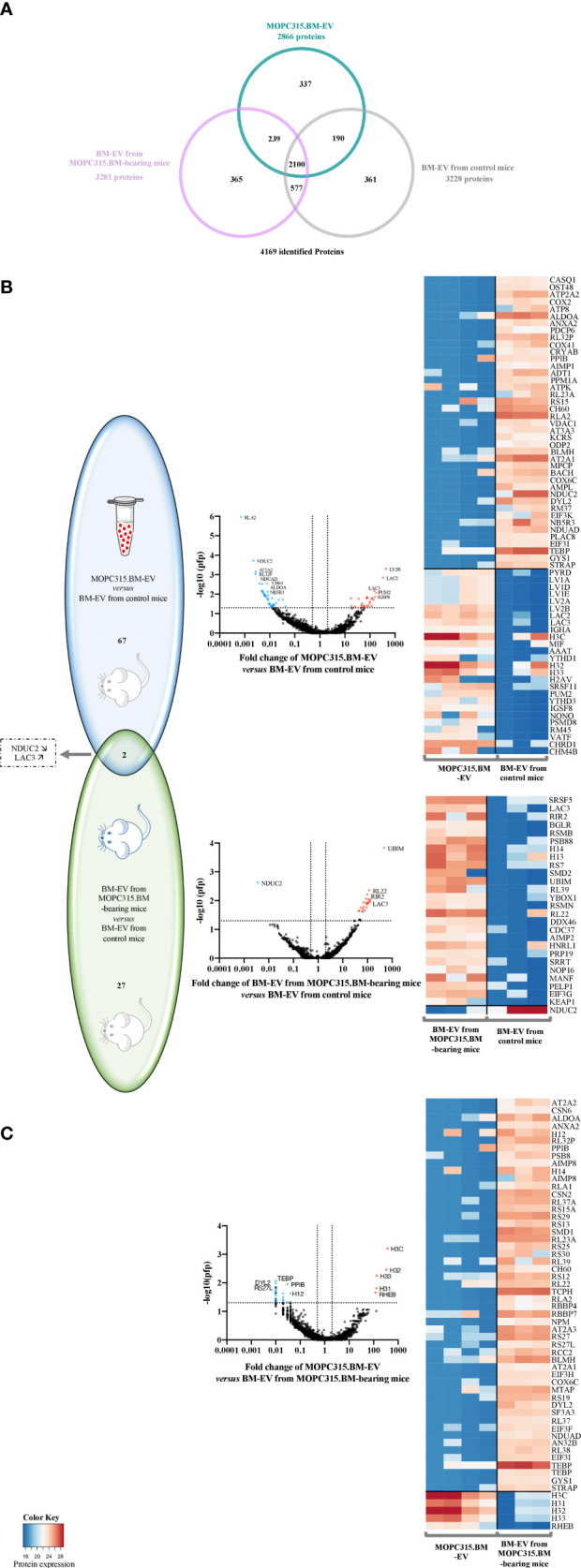
Proteomic analysis of differently expressed proteins from EV samples. **(A)** Venn diagram of the 4,169 identified proteins. Sample groups analyzed by mass spectrometry included EV from MOPC315.BM cell line (*n* = 4; blue) and EV from both BM of MOPC315.BM-bearing mice (*n* = 3; pink) and control mice (*n* = 3; gray). **(B)** Venn diagram (left) and respective volcano plots (middle) of differentially expressed proteins between MOPC315.BM-derived EV *versus* BM-EV from control mice (top; blue); and between BM-derived EV from MOPC315.BM-bearing mice *versus* control mice (bottom; green). **(C)** Venn diagram (left) and respective volcano plots (middle) of differentially expressed proteins between MOPC315.BM-derived EV *versus* BM-EV from MOPC315.BM-bearing mice. Proteins above the horizontal dotted line are those that met the selection criterion by false-positive prediction (pfp), as they present a pfp ≤ 0.05. The vertical dotted lines delimit the proteins whose fold change (FC) is equal or inferior to 0.5 (left line) and equal or superior to 2 (right line). The proteins highlighted in blue or red meet both requirements: pfp ≤ 0.05 and FC ≤ 0.5 (blue) or pfp ≤ 0.05 and FC ≥ 2 (red). Functional enrichment analysis of differentially expressed proteins was conducted using the FunRich software (UniProt database). On the right side is the corresponding heatmap representation of the protein expression profile of EV from MOPC315.BM cells versus BM-EV from control mice (top), and BM-derived EV from MOPC315.BM-bearing mice versus BM-derived EV from control mice (bottom).

We further explored the proteomic content of EV using mass spectrometry. Analysis of the proteomic signature in a total of 4,169 identified proteins was performed in EV isolated from the MOPC315.BM cell line (*n* = 4) and in EV recovered from the BM supernatant of both control (*n* = 3) and MOPC315.BM-bearing mice (*n* = 3) (**Figure 4A**).

This analysis revealed that 67 proteins are differently expressed between MOPC315.BM-derived EV versus EV from the BM of control mice. Gene set enrichment analysis determined an enhancement in biological processes involving necrotic cell death and calcium ion homeostasis and transport. The most upregulated proteins in EV from MOPC315.BM cells are the Ig lambda-2 chain V region MOPC 315 (LV2B), immunoglobulin lambda constant 2 (LAC2; *Iglc2* gene), and LAC3 (*Iglc3* gene). In parallel, we found 27 proteins that are differently expressed between EV from MOPC315.BM-bearing mice and control mice. These proteins are enriched in biological processes related to RNA splicing and protein translation. Within those, the only significantly decreased protein is the NADH dehydrogenase [ubiquinone] 1 subunit C2 (NDUC2; *Ndufc2* gene). The most upregulated protein between EV from MOPC315.BM-bearing mice and control mice is the ubiquitin-like protein FUBI (UBIM, *Fau* gene). Moreover, quantitative analysis also suggests that the 25 proteins differentially expressed between EV from MOPC315.BM-bearing mice versus control mice, but not differentially expressed between MOPC315.BM-EV compared to control mice, may be related to non-MOPC315.BM cells within the BM niche. In fact, total EV were recovered from the BM of mice and not isolated myeloma-derived EV. Of note, only 2 proteins are significantly deregulated in both EV from MOPC315.BM cells and MOPC-315.BM-bearing mice versus EV from control mice: NDUC2 and LAC3. The downregulated NDUC2 protein is involved in the mitochondrial electron transport, inflammatory response, negative regulation of NF-κB signaling, and necrosis. In contrast, the upregulated protein LAC3 is involved in B-cell and complement activation, phagocytosis, and innate immune response (**Figure 4B**). Furthermore, we performed a functional analysis of MOPC315.BM-derived EV versus the EV from MOPC315.BM-bearing mice. This analysis revealed 5 proteins upregulated in EV isolated from the MOPC315.BM cell line compared to EV from MOPC315.BM-bearing mice, namely, histone H3.3 type c (H3C; *H3f3c* gene) and Ras homolog, MTORC1 binding (RHEB*; Rheb* gene). These proteins are mainly involved in DNA replication-dependent nucleosome assembly and gene silencing. By contrast, there are 52 proteins downregulated in MOPC315.BM-EV compared to EV from MOPC315.BM-bearing mice, which are mainly involved in translation and ribosomal small subunit assembly (**Figure 4C**).

## Discussion

Despite overall survival improvement with available treatments, almost 50% of MM patients succumb to the disease within 5 years after diagnosis (34), reinforcing the need for more effective therapeutic approaches. Impaired immune surveillance is an emerging hallmark of MM, promoting the establishment, maintenance, and expansion of the tumor cells (3, 4). The disruption in immune homeostasis includes signs of exhaustion with sustained expression of inhibitory checkpoints, such as PD-1 or TIGIT by T cells (34, 35). Given this deregulation within the MM BM microenvironment, the search for agents enhancing anti-tumoral immunity has increased in recent years (36, 37).

To functionally explore the BM lymphoid compartment upon MM establishment, we performed an unbiased analysis of multiparametric immunophenotypic data. MOPC315.BM-bearing mice mainly showed a suppressive microenvironment compared to control mice, supporting that myeloma cells can control and reprogram the immune response, promoting myeloma cell survival and proliferation. For instance, it was previously shown that an increase in the expression of the IC PD-1 by lymphocytes is a mechanism of immune escape by myeloma cells in MM patients (3, 4). In this study, we also observed an increase in PD-1 expression by CD4+, CD8+ T cells and NKp46 cells in MOPC315.BM-bearing mice, corroborating the findings in MM patients. Similarly, we also found an increase in the expression of CTLA-4 by yδ+ T cells in MOPC315.BM-bearing mice compared to controls. This inhibitory molecule is rarely expressed in the surface of γδ + T cells (38), but it was previously reported to be associated with a poorer prognosis in childhood acute leukemia (39). Also, a decrease in the expression of CD25 by CD4+ T cells and CD27 by yδ + T cells might be related to a decrease in the effector functions of these populations. No statistical differences were found in the expression of CD44 between the controls and the experimental groups (data not shown). A clear distinction of the CD3+ subpopulation of Nkp46- γδ- CD4- CD8- cells present in cluster #6 was not possible; nonetheless, others have already described the existence of CD3 double-negative populations (40).

On the other hand, gene expression analysis revealed that CD8+ and CD4+ T cells are producing *Gzmb* and *Prf1*, indicating their functional activation, or are producing *Ctla4* and *Il10*, indicating suppression. In particular, *Il10* is known to have anti-inflammatory properties and the production of this cytokine by lymphocytes suggests a mechanism of immune escape in MM. Indeed, Plaumann et al. showed that IL-10 favors the generation and expansion of a specific group of suppressive CD8+ Tregs (CD8^+^CD28^-^CD57^+^LFA-1^high^), leading to an inhibition of antigen-specific T-cell responses in MM (41). Interestingly and although in solid malignancies, IL-10 was also shown to increase the expression of other negative receptors associated with suppression in CD8+ T cells, namely, PD-1, Lag-3, or TIGIT (42, 43).

These results hint at the presence of different niches where tumor may remain stable or, on the contrary, may progress. This might be related to myeloma intra-clonal heterogeneity. However, the presence of immune cell populations with anti-tumor activity does not preclude MM development. Overall, these immune alterations reported in this manuscript endorse the potential efficacy of combinatory approaches of immunotherapy as a way to increase or stimulate the immunity against MM tumor cells.

Previous results suggest that myeloma-derived EV play an important role in the modulation of the BM milieu (44). Here, the analysis of the BM microenvironment of conditioned mice with MOPC315.BM-derived EV revealed a significant modulation of the phenotype of lymphocytes, establishing a suppressive milieu. CD4+ T cells exhibited a pro-tumorigenic profile with higher expression of the IC PD-1 and CTLA-4 in conditioned mice compared to controls. Altogether, these data suggest that the presence of MOPC315.BM-derived EV leads to suppression of anti-tumor immunity by CD4+ T cells, even in the absence of MM clonal cells. Interestingly, a similar increase in the expression of the IC marker PD-1 by CD4+ T cells was induced in MOPC315.BM-bearing mice. Increased expression of PD-1-expressing CD4+ T cells in MM patients compared to healthy donors has been previously reported (45, 46), but never by induction of MM-derived EV as we show in this work. Moreover, in MOPC315.BM-bearing mice previously conditioned with MOPC315.BM-derived EV, an increase in CD27+ CD25+ CD4+ T cells was found, which, with the augment in PD-1, suggest an increased suppression.

Accordingly, the IC blockers anti-PD-1, -PD-L1, or -CTLA-4 monoclonal antibodies should be further explored as therapeutic agents against MM. A recent report by Kwon et al. showed that a combination of an anti-PD-1 monoclonal antibody with a TGF-β inhibitor was able to re-establish anti-tumor potential of CD8+ T cells (47). Although a phase III clinical trial combining anti-PD-1 monoclonal antibody with lenalidomide plus dexamethasone stopped due to toxicity, the follow-up time was 6.6 months, which was too short to understand the possible efficacy of this triple combination (48). This supports that further work is required, particularly in defining the best possible combination triggering tumor control with limited side effects.

The proteomic data displayed that both LV2B and LAC2/3 are upregulated in MOPC315.BM-derived EV, which is related to the intrinsic genomic properties of MOPC315.BM cells. Indeed, this finding is in line with the previous description of the Ig lambda immunotype of MOPC315 cells, the parental cell line of MOPC315.BM cells (31, 32, 49–53). Interestingly, the sarcoplasmic/endoplastic reticulum calcium ATPase 1 and 3 (ATP2A1 and ATP2A3), which are involved in the biological process of apoptosis, are downregulated in EV from MOPC315.BM cells when compared to EV from control mice. This is consistent with the increased survival distinguished in myeloma cells.

Our results also showed that the UBIM protein is upregulated in EV from MOPC315.BM-bearing mice when compared to control mice. The exact functions of this protein are not fully understood, and its role in cancer has been debated (54, 55). Nonetheless, UBIM is part of the family of ribosomal proteins and a recent paper from Dabbah et al. demonstrated that the protein cargo of microvesicles from MM mesenchymal stromal cells had higher levels of ribosomal proteins compared to controls. Moreover, by restricting the levels of ribosomal proteins, the researchers found that MM proliferation was dependent on this cargo (56). Thus, the therapeutic targeting of the UBIM protein deserves further investigation. By contrast, the metabolic regulator NDUC2 is downregulated in myeloma EV. Using MM cell lines and patient samples, this protein was shown to be a biomarker of Venetoclax sensitivity (57) and to be associated with a negative regulation of the non-canonical NF-κB pathway (58). Furthermore, this signaling pathway is shown to be involved in the upregulation of IC expression by tumor cells (e.g., PD-L1 upregulation), resulting in impaired immune response by T cells (59–63). Interestingly, the protein cargo of EV from the cell line MOPC315.BM and EV obtained from MOPC315.BM-bearing mice were mostly involved in the regulation of gene silencing and DNA replication, which is in accordance with the neoplastic origin of these plasma cells. One example is the functional enrichment we found in the RHEB protein and its involvement in the mTOR signaling pathway, which is known to be dysregulated in MM (64).

The proteomic content of MOPC315.BM-derived EV may interfere with immune cell functions, favoring myeloma cells. Indeed, our group recently showed that the proteomic content of MM-derived EV is mainly involved in immunosuppression, strongly suggesting that these EV are promoters of tumor-mediated immunosuppression (19). This phenomenon can happen through several pathways. Besides their intracellular content, EV also express several markers in their surface, namely, MHC receptors. Considering that lymphocytes are not specialized in antigen uptake, we hypothesize that T cells may interact with surface molecules present in EV to deliver signals that result in the activation of downstream signaling pathways, thus leading to changes in recipient cells, inducing their polarization to tumor-promoting phenotypes (65). Another possible importance of EV on MM could be the expression of CD38 in EV from MM cells, somehow explaining the resistance to anti-CD38 monoclonal antibody-based treatment. In this case, the drug would bind to the circulating EV and less to the myeloma cells (66).

Taken together, we have shown that MM-derived EV promote immunosuppression by modulating the phenotype of lymphoid cells with an increase in the expression of the IC PD-1 and CTLA-4, accompanied by a decrease in CD27 expression, thus facilitating myeloma cell escape and progression. Consequently, manipulating MM-derived EV action on the BM microenvironment, considering all the immune alterations reported in this work, should be further investigated as a new therapeutic tool for MM treatment.

Overall, the data presented here lay ground the foundation for further studies modulating the tumoral microenvironment aiming at MM patient outcome improvement.

## Data Availability Statement

The data supporting the conclusions of this article will be made available by the authors upon request, without undue reservation. The datasets generated from mass spectrometry (proteomic), can be found in the ProteomeXChange (67) Consortium via the PRIDE (68) partner repository. This data can be found here: https://www.ebi.ac.uk/pride/ under the accession number PXD032934.

## Ethics Statement

The animal study was reviewed and approved by the Institutional Ethical Committee - Champalimaud Animal Welfare Body (ORBEA), Champalimaud Foundation, Lisbon, Portugal; National Competent Authority - Portuguese Directorate-General for Animal Welfare (DGAV), Lisbon, Portugal.

## Author Contributions

RL, EC, BF, and CJ conceptualized the study. All the authors performed the experimental research. RL and CP performed both supervised and unsupervised analysis. RL and CP performed the statistical analysis. RL, EC, and CJ wrote the original draft. CJ supervised the study. All authors participated in data discussion and manuscript revision. All authors contributed to the article and approved the submitted version.

## Funding

This work was supported by Portuguese national funds, through FCT—*Fundação para a Ciência e Tecnologia*—in the context of the projects PDTC/MEC-HEM/30315/2017 and PCDC/MED-ONC/1215/2021, and by GILEAD GÉNESE (project PGG/058/2019). RL received a fellowship funded by FCT (2020.4875.BD). This work was also supported by the research infrastructure CONGENTO, co-financed by Lisboa Regional Operational Programme (Lisboa2020), under the PORTUGAL 2020 Partnership Agreement, through the European Regional Development Fund (ERDF) and FCT under the project LISBOA-01-0145-FEDER-022170. Fundings were not involved in the study design, collection, analysis, interpretation of data, writing of the article, or decision to submit it for publication.

## Conflict of Interest

The authors declare that the research was conducted in the absence of any commercial or financial relationships that could be construed as a potential conflict of interest.

## Publisher’s Note

All claims expressed in this article are solely those of the authors and do not necessarily represent those of their affiliated organizations, or those of the publisher, the editors and the reviewers. Any product that may be evaluated in this article, or claim that may be made by its manufacturer, is not guaranteed or endorsed by the publisher.

## Supplementary Material

Supplementary Figure 1 Illustrative flow cytometry gating strategy used to identify lymphoid populations and their phenotype.
