# Supplementary figures and images for: Multiple Myeloma-Derived Extracellular Vesicles Modulate the Bone Marrow Immune Microenvironment
Source: Front Immunol. 2022 Jul 7;13:909880. doi: 10.3389/fimmu.2022.909880 (PMC9302002; doi:10.3389/fimmu.2022.909880)

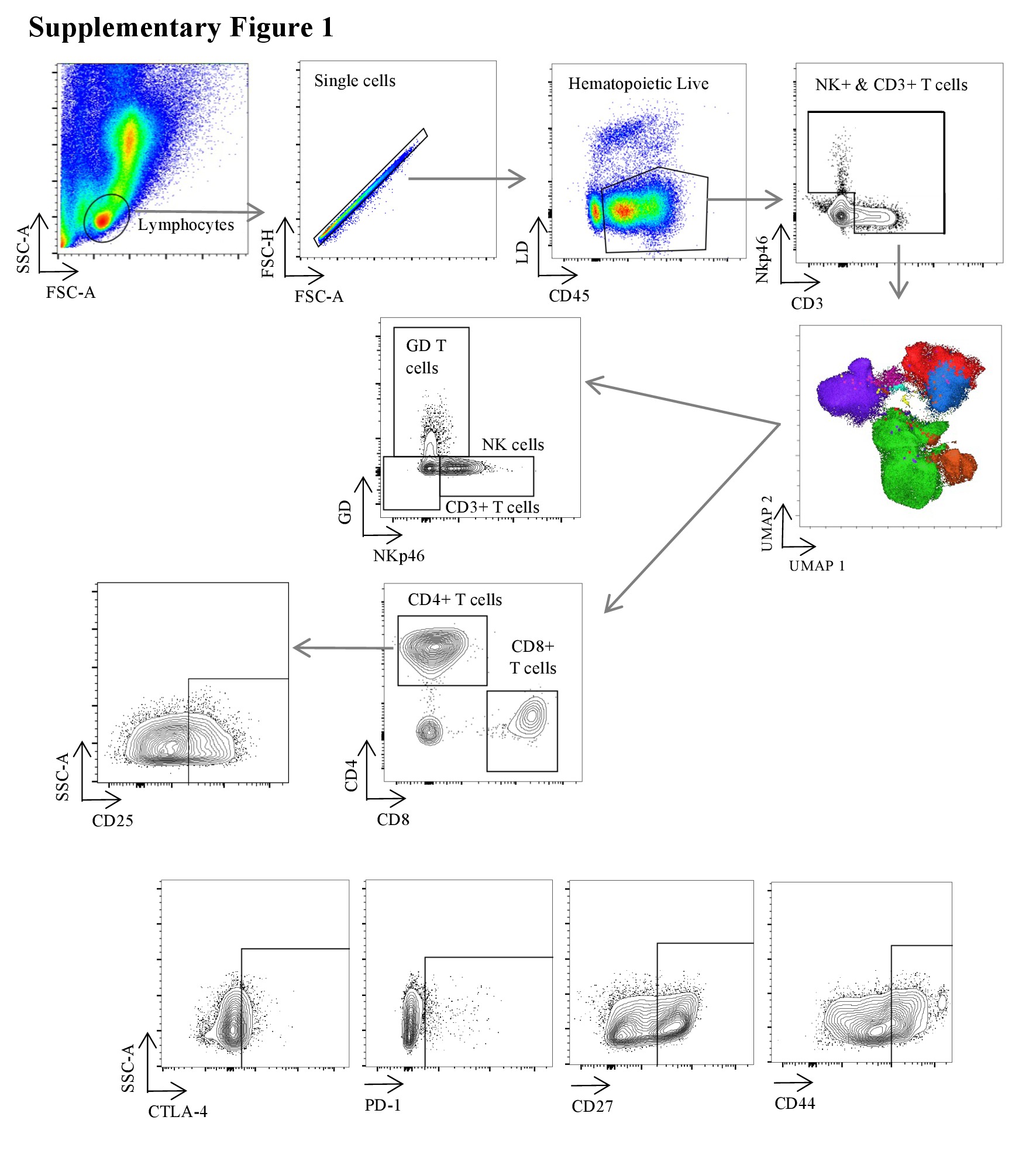

Supplement: Supplementary Figure 1 — Illustrative flow cytometry gating strategy used to identify lymphoid populations and their phenotype. [file Image_1.jpeg]
